# Inverse Associations Between Circulating Secreted Frizzled Related Protein 2 (sFRP2) and Cardiometabolic Risk Factors

**DOI:** 10.3389/fcvm.2021.723205

**Published:** 2021-10-14

**Authors:** Mengying Cao, Hao Wang, Wenshu Li, Xueli Jiang, Xiaolin Wang, Wei Guo, Pan Gao, Yunzeng Zou

**Affiliations:** ^1^Shanghai Institute of Cardiovascular Diseases, Shanghai Clinical Bioinformatics Research Institute, Zhongshan Hospital, Fudan University, Shanghai, China; ^2^Department of Laboratory Medicine, Zhongshan Hospital, Fudan University, Shanghai, China; ^3^School of Public Health, Key Lab of Public Health Safety of the Ministry of Education and Key Lab of Health Technology Assessment of the Ministry of Health, Fudan University, Shanghai, China

**Keywords:** secreted frizzled-related protein 2 (sFRP2), cardiometabolic risk factors, heart failure (HF), type 2 diabetes (T2DM), cross-section study

## Abstract

**Background:** Secreted frizzled-related protein 2 (sFRP2) plays an important role in metabolic syndrome and cardiovascular diseases (CVDs); However, its relevance with cardiometabolic diseases remains to be elucidated. We aimed to determine the serum levels of sFRP2 in patients at different stages of heart failure (HF) with or without type 2 diabetes mellitus (T2DM), and assess the correlation between circulating sFRP2 levels and cardiometabolic risk factors.

**Methods:** In this study, serum samples from 277 patients visiting Zhongshan Hospital affiliated to Fudan University were collected. These patients were clinically diagnosed and categorized as five groups, including the control group, pre-clinical HF group, pre-clinical HF+T2DM group, HF group and HF+T2DM group. Serum sFRP2 levels were measured with enzyme-linked immunosorbent assay (ELISA) tests and the clinical characteristics of each patient were recorded. Spearman rank correlation analysis and multiple stepwise linear regression analysis were conducted. Univariate and multivariate logistic regression analysis were performed to screen risk factors for HF in patients with CVDs.

**Results:** Serum sFRP2 levels were significantly lower in the HF+T2DM group compared with the other four groups. Spearman rank correlation analysis showed that sFRP2 was negatively correlated with parameters including patients' age, fasting plasma glucose (FPG), glycated hemoglobin A1c (HbA1c), cardiac troponin T (cTNT), N-terminal pro-B-type natriuretic peptide (NT-proBNP), high-sensitivity C-reactive protein (hs-CRP), left atrial dimension (LAD) and left ventricular posterior wall (LVPW), and positively correlated with hemoglobin, estimated glomerular filtration rate (eGFR), albumin, total cholesterol (TC), low-density lipoprotein cholesterol (LDL-C), and left ventricular ejection fraction (LVEF). However, in multiple regression analysis, significant associations with ln(sFRP2) were observed only in FPG, hs-CRP and LAD. Higher serum sFRP2 was significantly linked to lower odds of HF in patients with CVDs.

**Conclusion:** sFRP2 progressively decreased when glucose homeostasis and cardiac function deteriorated. sFRP2 acted as a risk factor for HF in patients with CVDs, especially in those with concomitant T2DM.

## Introduction

Despite numerous advances in diagnosis and treatment, cardiovascular diseases (CVDs) remain the leading cause of global mortality. Heart failure (HF), characterized by dyspnea or limited activity due to fluid retention and/or reduced cardiac output, is the end stage of most CVDs and affects at least 23 million people worldwide (1). To emphasize the importance of early intervention and guide therapy, the American College of Cardiology Foundation/American Heart Association (ACCF/AHA) started to promote the concept of HF stages in 2001 (2) and have categorized HF into four stages. Stage A is defined as high risk for HF, including hypertension, metabolic syndrome, tobacco use, and known cardiotoxic agents. Stage B is defined as heart disease without signs or symptoms of HF. Stage C refers to HF with prior or current symptoms, and stage D refers to refractory HF requiring specialized interventions (3). The China Medical Association (CMA) has adapted this concept since 2007 and has classified HF into pre-HF, pre-clinical HF, clinical HF and refractory HF stages (4), which correspond to stages A to D in the American guideline, respectively. The progression of HF stages is irreversible and is associated with a decrease in 5-year survival (5). The rational for treating each stage of HF varies from controlling risk factors, treating structural heart disease to reducing morbidity and mortality.

In the past decade, the prevalence of type 2 diabetes (T2DM) has increased by 30% globally (6), with the number of influenced Chinese adults increasing from 128 million in 2007 to 155 million in 2017 (7). Previous studies have consistently demonstrated a 2- to 4-fold increased risk of HF in individuals with T2DM compared with their age- and sex-matched non-diabetic counterparts, even after adjustment for other cardiovascular risk factors (8, 9). The blood glucose control level also influences the development and progression of HF (10–12). Moreover, HF patients with concomitant T2DM exhibit significantly worse outcomes compared with those without T2DM (13). Therefore, investigating the T2DM-related characteristics in a HF patient cohort may give insight into our understanding of how the metabolic disorders incorporate with CVDs.

Secreted frizzled-related protein (sFRP) family consists of 5 members (sFRP1-5) that structurally resemble the Wnt frizzled receptors and play important roles in cardiac development and various cardiovascular pathophysiological conditions (14). Among these proteins, sFRP2 is considered to be the most potent modulator of the Wnt signaling (15). Previous studies have shown divergent roles of sFRP2 in myocardial fibrosis (16–18), hypertrophy (19), angiogenesis (20), cardiac cell death (21), and regeneration (22, 23) *via* bi-directional regulation of the canonical Wnt pathway, activation of non-canonical Wnt pathway or modulation of other signaling pathways (24). The expression of sFRP2 in cardiac tissues was increased in the early stage of HF and subsequently decreased with the progression of HF (19). In patients with severe decompensated HF, serum sFRP2 levels were higher in those experiencing primary outcome events (25). However, data on serum sFRP2 are still lacking in healthy subjects and in patients with cardiac abnormalities that have not developed into HF. As an adipokine, sFRP2 expression in the white adipose tissue (WAT) was upregulated in obesity (26) and downregulated with moderate weight loss (27). Serum sFRP2 was positively associated with circulating insulin, homeostasis model assessment of β cell secretory capacity (HOMA-β) and insulin resistance (HOMAIR), body mass index (BMI) and triglycerides (TG), and was increased in patients with abnormal glucose tolerance (AGT) (28). Circulating sFRP4 (29–32) and sFRP5 (33–36) levels were associated with the risk of prediabetes/T2DM, obesity and CVDs. Lower levels of serum sFRP5 was associated with worse prognosis in patients with HF, especially in those with concomitant T2DM (37). However, it is obscure whether sFRP2 is involved in the comorbidity of HF and T2DM.

In this study, we investigated the serum levels of sFRP2 among patients in different HF stages with or without T2DM and analyzed its association with fasting blood glucose (FBG), glycated hemoglobin (HbA1c), blood lipid levels, BMI and other cardiometabolic risk factors. We also explored whether sFRP2 could serve as an indicator of the comorbidity of HF and T2DM.

## Methods

### Study Population

This study complied with the principles of the Declaration of Helsinki and was approved by the Ethics Committee of Zhongshan Hospital, Fudan University (B2020-078R). During the period of August 2020 to August 2021, a total of 425 serum samples from patients admitted to Zhongshan Hospital were initially collected. Those with T1DM, history of malignancy, autoimmune diseases, severe infection, severe renal failure (estimated glomerular filtration rate (eGFR) <30 mL/min/1.73 m^2^ or under renal replacement therapy), severe hepatic disease (bilirubin > 3× the upper limit of normal, or aspartate aminotransferase/alanine aminotransferase/alkaline phosphatase > 5× the upper limit of normal, or cirrhosis), active bleeding or severe anemia (Hb < 60 g/L) were excluded from the study.

Finally, 277 of these serum samples were included for the following analysis. Patients were classified and grouped according to the Guideline for the diagnosis and treatment of heart failure in China (2018 edition) (38) and the guideline for T2DM (2020 edition) (39). The healthy subjects and pre-HF patients (stage A) were pooled as control group (*n* = 53) and none of them was diagnosed with T2DM. The pre-clinical HF patients (stage B) were subdivided into pre-clinical HF group without T2DM (*n* = 57) and pre-clinical HF+T2DM group (*n* = 54), depending on their medical history of T2DM. The HF patients, including both clinical HF (stage C) and refractory HF (stage D), were also stratified by the medical history of T2DM and thus subdivided into HF group without T2DM (*n* = 63) and HF+T2DM group (*n* = 50).

The cardiac conditions of these patients were further determined following the latest guidelines for hypertension (40), ischemic heart disease (41, 42), valvular heart disease (43), cardiomyopathy (44), and congenital heart disease (45). Based on echocardiography, patients with left ventricular ejection fraction (LVEF) <40%, 40–49%, and ≥50% were subclassified as HF with reduced ejection fraction (HFrEF), midrange EF (HFmrEF), and preserved EF (HFpEF), respectively (46).

### Clinical Characteristics and sFRP2 Detection

Medical records were carefully documented, including age, gender, BMI, smoking habit, history of hypertension and CVDs, medication, New York Heart Association (NYHA) functional class, biochemistry tests and echocardiographic parameters.

Fasting venous blood was collected with a serum separation tube, followed by stratification at room temperature for 15 min. Serum were separated and then stored at −80°C until analysis. An enzyme-linked immunosorbent assay (ELISA) kit purchased from Shanghai Yu Bo Biotech Co., Ltd was used to measure the levels of serum sFRP2 in accordance with the manufacturer's instruction manual. The intra-assay and inter-assay variations were 5.4% and 7.5%, respectively.

### Statistical Analysis

Statistical analyses were performed in R software (Version 4.0.4) and GraphPad Prism Software (Version 8.3.0). Continuous variables were presented as mean ± standard error of mean or median and interquartile range. Categorical variables were exhibited as numbers or proportions. Differences among groups were compared using one-way ANOVA test for normally distributed continuous variables with homogeneous variance, otherwise Kruskal–Wallis test along with Dunn *post-hoc* test were used. Pearson's chi-squared test or Fisher's exact test was used for categorical variables. Spearman rank correlation analysis identified factors correlated with sFRP2. The levels of sFRP2 were log_e_-transformed (ln-transformed) for further analyses to optimize the fitted equation. Cross-sectional associations between ln(sFRP2) and cardiometabolic risk factors were estimated by multiple stepwise linear regression analysis. Multicollinearity was considered present for a variance inflation factor (VIF) > 2.5. Univariate logistic regression analysis was performed to screen risk factors for HF and the variables with significance were further investigated in the multivariate logistic stepwise regression analysis. All statistical tests were two-sided and a *P*-value < 0.05 was considered as statistically significant.

## Results

### Clinical Characteristics of the Study Population

The 277 participants were divided into 5 groups: control group (*n* = 53), pre-clinical HF group (*n* = 57), pre-clinical HF+T2DM group (*n* = 54), HF group (*n* = 63) and HF+T2DM group (*n* = 50). There was no significant difference in demographic characteristics such as age, gender, BMI and smoking habit among these groups. The pre-clinical HF+T2DM group and HF+T2DM group had higher FPG and HbA1c than their nondiabetic counterparts and the control group (Table 1). Compared with the control group, the levels of cardiac troponin T (cTNT), N-terminal pro-B-type natriuretic peptide (NT-proBNP) and left atrial dimension (LAD) increased in the pre-clinical HF group and further elevated in the HF group (Tables 1, 2). Similar results were obtained in the pre-clinical HF+T2DM group and the HF+T2DM group. Patients with CVDs exhibited reduced LVEF and elevated pulmonary artery pressure (PAP) compared with the control group. Echocardiographic examinations showed enlarged left ventricular end-diastolic dimension (LVDd) and left ventricular end-systolic dimension (LVDs), as well as thickened interventricular septal thickness (IVS) and left ventricular posterior wall (LVPW) in the HF group and HF+T2DM group compared with the control group, pre-clinical HF group and pre-clinical+T2DM group (Table 2).

**Table 1:** Demographic and laboratory information of the study population.

	**Control (*n* = 53)**	**Pre-HF (*n* = 57)**	**Pre-HF +T2DM (*n* = 54)**	**HF (*n* = 63)**	**HF + T2DM (*n* = 50)**	***P*-value**
**Demographic characteristics**						
Age (years)	62 (56, 66)	65 (54, 67)	66 (56, 71)	64 (52, 72)	68 (62, 74)	0.15
Gender (male%)	32 (60.4)	40 (70.2)	41 (75.9)	46 (73.0)	31 (62.0)	0.33
BMI (kg/m^2^)	23.8 ± 0.6	24.2 ± 0.5	24.8 ± 0.4	25.1 ± 0.6	24.7 ± 0.6	0.16
Smoking [n (%)]	15 (28.3)	19 (33.3)	19 (35.1)	17 (27.0)	8 (18.0)	0.22
**Biochemistry indicators**						
Hemoglobin (g/L)	142.8 ± 1.6	130.0 ± 2.5	128.7 ± 2.5	121.0 ± 1.9	111.0 ± 3.9	*<0.001*
Alb (g/L)	46.8 ± 0.5	40.8 ± 0.5	40.5 ± 0.7	38.4 ± 0.6	36.9 ± 0.7	*<0.001*
eGFR (mL/min/1.73 m^2^)	87.3 ± 1.9	83.6 ± 2.2	77.6 ± 2.7	75.3 ± 3.0	62.8 ± 3.8	*<0.001*
FPG (mmol/L)	4.9 (4.6, 5.1)	5.5 (4.9, 6.1)	6.6 (5.6, 8.2)	5.4 (4.7, 6.4)	8.5 (6.1, 10.6)	*<0.001*
HbA1c (%)	5.7 (5.5, 5.9)	5.6 (5.4, 5.8)	6.6 (5.9, 7.8)	5.9 (5.4, 6.4)	7.6 (6.4, 8.1)	*<0.001*
cTNT (ng/mL)	0.005 (0.004, 0.006)	0.019 (0.007, 0.077)	0.015 (0.009, 0.056)	0.082 (0.025, 0.192)	0.055 (0.028, 0.142)	*<0.001*
NT-proBNP (pg/mL)	36.3 (27.5, 43.4)	310.0 (64.6, 698.0)	284.0 (77.1, 1410.0)	1263.0 (676.0, 2759.0)	2261.0 (647.0, 5270.0)	*<0.001*
TC (mmol/L)	4.97 (4.47, 5.48)	3.32 (2.88, 3.88)	3.63 (2.98, 4.88)	3.76 (3.14, 4.26)	3.32 (2.83, 3.93)	*<0.001*
TG (mmol/L)	1.47 (1.10, 2.21)	1.33 (0.94, 1.70)	1.58 (1.02, 2.03)	1.25 (0.93, 1.87)	1.51 (1.15, 2.12)	0.09
LDL-C (mmol/L)	2.82 (2.46, 3.32)	1.56 (1.26, 2.17)	1.64 (1.32, 2.56)	2.01 (1.57, 2.62)	1.56 (1.25, 2.02)	*<0.001*
HDL-C (mmol/L)	1.24 (1.08, 1.48)	1.08 (0.86, 1.31)	1.08 (0.83, 1.31)	0.99 (0.85, 1.13)	0.98 (0.80, 1.16)	*<0.001*
hs-CRP (mg/L)	1.0 (0.4, 1.3)	1.0 (0.1, 2.8)	1.4 (0.6, 5.4)	3.8 (1.0, 8.8)	3.5 (1.2, 26.2)	*<0.001*

*Continuous variables are presented as mean ± standard error of mean or median (interquartile range). Categorical variables are expressed as number (percentages). Statistically significant values are indicated in italic. BMI, body mass index; Alb, albumin; eGFR, estimated glomerular filtration rate; FPG, fasting plasma glucose; HbA1c, glycated hemoglobin; cTNT, cardiac troponin T; NT-proBNP, N-terminal pro-B-type natriuretic peptide; TC, total cholesterol; TG, triglyceride; LDL-C, low-density lipoprotein cholesterol; HDL-C, high-density lipoprotein cholesterol; hs-CRP, high-sensitivity C-reactive protein*.

**Table 2:** Medical history and echocardiography of the study population.

	**Control (*n* = 53)**	**Pre-HF (*n* = 57)**	**Pre-HF+T2DM (*n* = 54)**	**HF (*n* = 63)**	**HF+T2DM (*n* = 50)**	***P*-value**
**History of cardiovascular diseases**						
IHD [n (%)]	/	47 (82.6)	51 (94.4)	30 (47.6)	27 (54.0)	*<0.001*
VHD [n (%)]	/	4 (7.0)	6 (11.1)	19 (30.2)	14 (28.0)	*<0.01*
Cardiomyopathy [n (%)]	/	2 (3.5)	2 (3.7)	8 (12.7)	5 (10.0)	0.16
CHD [n (%)]	/	3 (5.3)	3 (5.6)	6 (9.5)	3 (6.0)	0.83
Hypertension [n (%)]	19 (35.8)	33 (57.9)	45 (83.3)	27 (42.9)	40 (80.0)	*<0.001*
**Chronic medication**						
ACEI/ARBs [n (%)]	12 (22.6)	16 (28.1)	17 (31.5)	14 (22.2)	18 (36.0)	0.45
CCBs [n (%)]	11 (20.8)	15 (26.3)	17 (31.5)	13 (20.6)	22 (44.0)	*<0.01*
Beta-blockers [n (%)]	1 (1.9)	28 (49.1)	22 (40.7)	20 (31.7)	21 (42.0)	*<0.001*
Diuretics [n (%)]	3 (5.7)	6 (10.5)	7 (13.0)	29 (46.0)	19 (38.0)	*<0.001*
Digoxin [n (%)]	/	0 (0.0)	1 (1.9)	7 (11.1)	4 (8.0)	*<0.05*
Nitrate esters [n (%)]	/	8 (14.0)	11 (20.4)	11 (20.7)	16 (32.0)	0.12
Antiplatelet [n (%)]	/	40 (70.2)	28 (51.9)	31 (49.2)	28 (56)	0.10
Anticoagulant [n (%)]	/	9 (15.8)	3 (5.6)	15 (23.8)	4 (8.0)	*<0.05*
Insulin [n (%)]	/	/	9 (16.7)	/	17 (34.0)	*<0.05*
OAD [n (%)]	/	/	49 (90.7)	/	19 (38.0)	*<0.001*
Statins [n (%)]	7 (13.2)	33 (57.9)	19 (35.2)	13 (20.6)	24 (48.0)	*<0.001*
**Echocardiographic parameters**						
LVEF (%)	67 (63,69)	62 (56, 66)	63 (57, 66)	60 (35, 64)	58 (44, 65)	*<0.001*
ARD (mm)	33 (30, 34)	34 (30, 36)	33 (31, 36)	33 (31, 37)	34 (32, 38)	0.17
LAD (mm)	36 (33, 38)	40 (37, 43)	41 (38, 44)	47 (41, 51)	44 (42, 48)	*<0.001*
LVDd (mm)	46 (44, 47)	46 (44, 51)	47 (44, 53)	53 (46, 58)	51 (47, 59)	*<0.001*
LVDs (mm)	30 (28, 30)	30 (29, 34)	30 (28, 35)	35 (30, 47)	32 (30, 43)	*<0.001*
IVS (mm)	9 (8, 10)	10 (9,11)	10 (9, 13)	10 (9, 10)	10 (9, 10)	*<0.001*
LVPW (mm)	8 (8, 9)	9 (9, 10)	9 (9, 10)	10 (9, 11)	10 (9, 11)	*<0.01*
PAP (mmHg)	30 (29, 30)	31 (30, 35)	33 (31, 38)	36 (31, 44)	35 (31, 45)	*<0.001*

*Continuous variables are presented as mean ± standard error of mean or median (interquartile range). Categorical variables are expressed as number (percentages). Statistically significant values are indicated in italic. IHD, ischemic heart disease; VHD, valvular heart disease; CHD, congenital heart disease; ACEI/ARB, angiotensin-converting enzyme inhibitors or angiotensin II receptor blockers; CCB, calcium channel blocker; OAD, oral antidiabetic drugs; LVEF, left ventricular ejection fraction; ARD, aortic root dimension; LAD, left atrial dimension; LVDd, Left ventricular end-diastolic dimension; LVDs, left ventricular end-systolic dimension; IVS, interventricular septal thickness; LVPW, left ventricular posterior wall; PAP, pulmonary artery pressure*.

### Serum sFRP2 Levels in Different Groups

**Figure 1:**
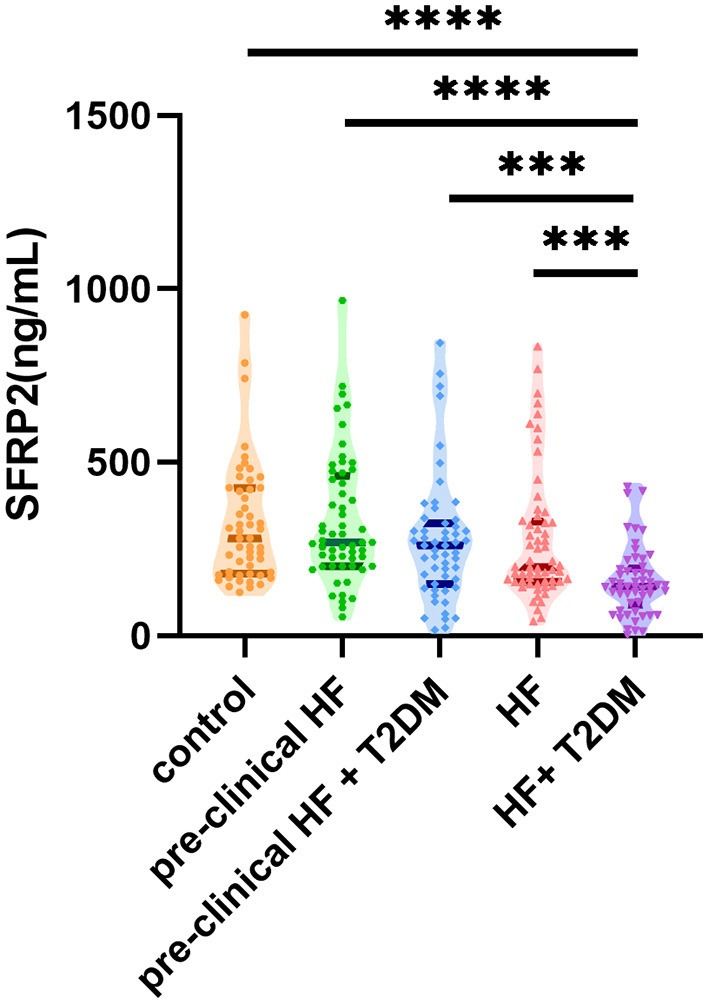
Serum sFRP2 in different groups (***P < 0.001, ****P < 0.0001).

Next, we compared the serum sFRP2 levels among the patient groups (Figure 1). There was no significant difference between the control group and the pre-clinical HF group (*P* = 0.94) or the HF group (*P* = 0.43). However, compared with the control group, serum sFRP2 level decreased significantly in the HF+T2DM group (*P* <0.0001). Although no significant difference was found between the pre-clinical HF group and the pre-clinical HF+T2DM group (*P* = 0.62), the HF+T2DM group had lower levels of serum sFRP2 than the HF group (*P* <0.0001). Our results showed that serum sFRP2 levels reduced in the co-occurrence of HF and T2DM, rather than in either individual condition.

### Association of sFRP2 With Clinical Variables

The correlation between sFRP2 and clinical variables were analyzed with Spearman rank correlation analysis using data from all the 277 participants. The presence and absence of diseases were assigned as 1 and 0, respectively. No, past and current tobacco use were assigned as 0,1,2, respectively. The detailed data are shown in Table 3. The Spearman correlation coefficient between sFRP2 and FPG was <-0.5, suggesting a moderate inverse correlation. SFRP2 had weak negative correlations with HbA1c, cTNT, NT-proBNP and weak positive correlations with hemoglobin and eGFR, as evidenced by the coefficients between −0.4 and −0.2 or 0.2 and 0.4. Although the *P*-values were <0.05, the correlation coefficients of sFRP2 with age, albumin, TC, LDL-C, high-sensitivity C-reactive protein (hs-CRP), LVEF, LAD and LVPW were between −0.2 and 0.2, indicating that the correlations were marginal.

**Table 3:** Spearman's correlation of sFRP2 with clinical variables.

**Indicator**	**ρ**	***P*-value**
Age	−0.1591595	*<0.01*
Gender	−0.0567796	0.35
BMI	−0.0980334	0.16
Smoking	−0.0538868	0.38
IHD	−0.0252443	0.68
VHD	−0.0699490	0.25
Hypertension	−0.1141791	0.06
Hemoglobin	0.2127307	*<0.001*
Alb	0.1946989	*<0.01*
eGFR	0.2082241	*<0.001*
FPG	−0.5345865	*<0.001*
HbA1c	−0.3071817	*<0.001*
cTNT	−0.2118334	*<0.001*
NT-proBNP	−0.3009578	*<0.001*
TC	0.1492633	*<0.05*
TG	−0.0672510	0.29
LDL-C	0.1639592	*<0.01*
HDL-C	0.1214094	0.06
hs-CRP	−0.1856972	*<0.01*
LVEF	0.1175641	*<0.05*
ARD	−0.0618573	0.32
LAD	−0.1438706	*<0.05*
LVDd	−0.1023572	0.10
LVDs	−0.116702	0.06
IVS	−0.093653	0.13
LVPW	−0.1213423	*<0.05*
PAP	−0.0072189	0.91

*Statistically significant values are indicated in italic. BMI, body mass index; IHD, ischemic heart disease; VHD, valvular heart disease; Alb, albumin; eGFR, estimated glomerular filtration rate; FPG, fasting plasma glucose; HbA1c, glycated hemoglobin; cTNT, cardiac troponin T; NT-proBNP, N-terminal pro-B-type natriuretic peptide; TC, total cholesterol; TG, triglyceride; LDL-C, low-density lipoprotein cholesterol; HDL-C, high-density lipoprotein cholesterol; hs-CRP, high-sensitivity C-reactive protein; LVEF, left ventricular ejection fraction; ARD, aortic root dimension; LAD, left atrial dimension; LVDd, Left ventricular end-diastolic dimension; LVDs, left ventricular end-systolic dimension; IVS, interventricular septal thickness; LVPW, left ventricular posterior wall; PAP, pulmonary artery pressure*.

**Figure 2:**
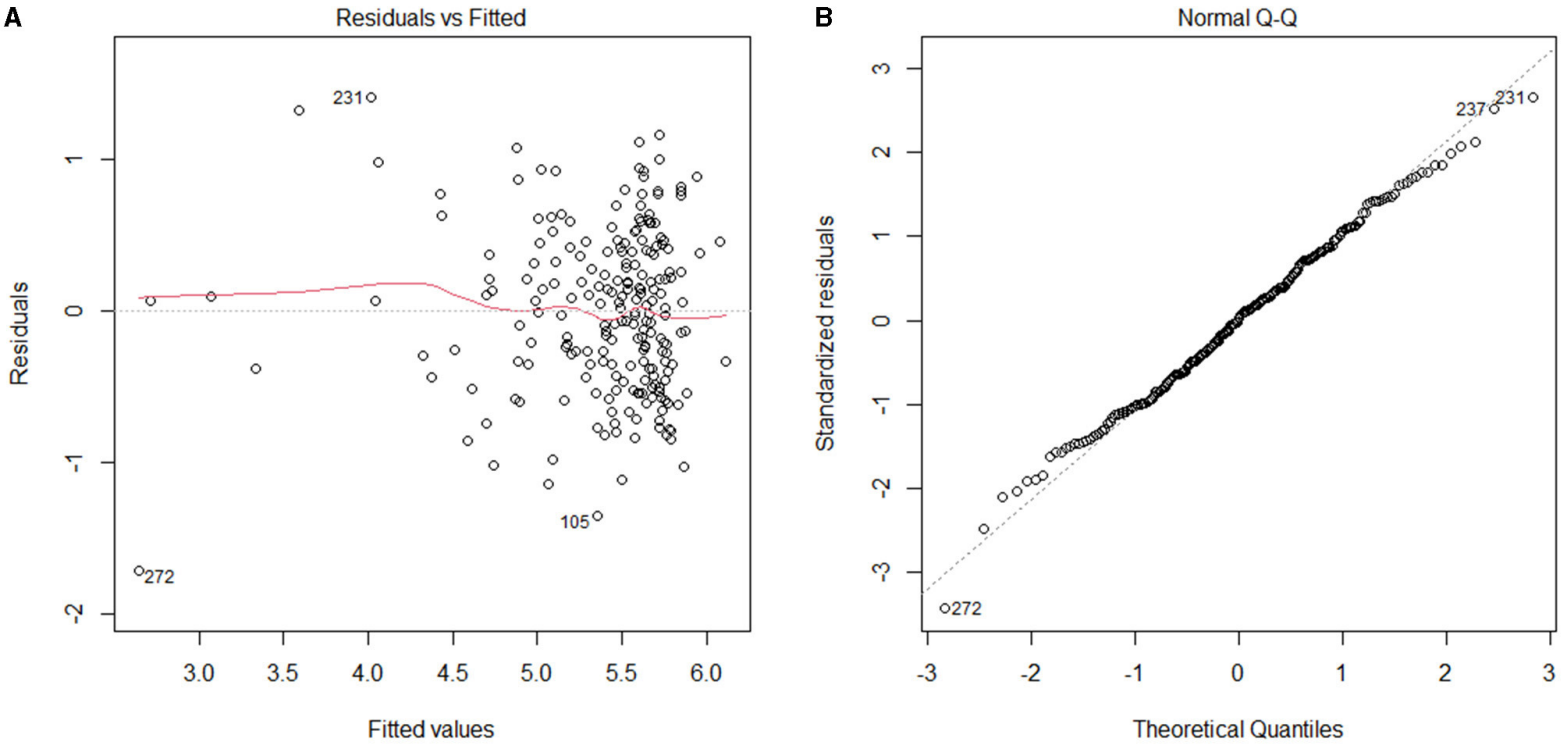
Residuals of the fully adjusted model. **(A)** Residuals vs. Fitted; **(B)** Normal QQ.

With ln(sFRP2) serving as the dependent variable and the clinical variables correlated with sFRP2 in Spearman correlation analyses as independent variables, a multiple stepwise regression analysis was performed using data from all the 277 participants. During the stepwise regression, age, hemoglobin, Alb, eGFR, HbA1c, cTNT, NT-proBNP, LDL-C and LVPW were removed to reach the lowest Akaike information criterion (AIC) level. Potential confounders such as medications (with or without drugs were assigned as 1 and 0, respectively) were also added as independent variables, but no significant associations were found (data not shown). Finally, FPG, TC, hs-CRP, LVEF and LAD were selected as independent variables in the optimized model. The *P*-value of the model was <2.2e-16 and the adjusted *R*-squared was 0.5109, which indicated 51.09% of the changes in ln(sFRP2) could be attributed to these factors (Table 4). VIFs were <1.5 in all analyses, suggesting that no collinearity was observed. As shown in Figure 2, residuals were equally distributed and approximately accorded with a normal distribution.

**Table 4:** Multiple regression analysis of ln(sFRP2) and clinical variables.

	**Estimate**	**Std. error**	**t value**	***P*-value**
(Intercept)	7.361267	0.414689	17.751	*<0.001*
FPG	−0.179817	0.015973	−11.258	*<0.001*
TC	0.058419	0.030584	1.910	0.06
hs-CRP	−0.006420	0.001744	−3.682	*<0.001*
LVEF	0.005843	0.003451	−1.693	0.07
LAD	−0.014792	0.006302	−2.347	*<0.05*

*Statistically significant values are indicated in italic. FPG, fasting plasma glucose; TC, total cholesterol; hs-CRP, high-sensitivity C-reactive protein; LVEF, left ventricular ejection fraction; LAD, left atrial dimension*.

### Relationship Between sFRP2 and the Risk of HF

To investigate the risk factors of HF in patients with CVDs, a univariate logistic regression analysis was performed between pre-clinical HF patients (the pre-clinical HF group and pre-clinical HF+T2DM group) and HF patients (the HF group and HF+T2DM group). In total, 224 of the 277 participants were included in this analysis. As shown in Table 5, hemoglobin, albumin, eGFR and ln(sFRP2) were negatively associated with the odds of HF, whereas FPG, HbA1c, and hs-CRP showed positive associations with the odds of HF. The 7 variables with significance were further investigated in the multivariate logistic stepwise regression analysis. Finally, hemoglobin, albumin, eGFR and ln(sFRP2) were included in the logistic regression equation (Table 6).

**Table 5:** Risk factors for HF in 244 cardiac patients: univariate logistic regression.

	**OR (95% CI)**	***P*-value**
Age	1.0166780 (0.9958878, 1.0379020)	0.12
Gender	0.8284024 (0.4743534, 1.4467070)	0.51
BMI	1.0403330 (0.9532089, 1.1354200)	0.38
Smoking	0.7870194 (0.5334641, 1.1610890)	0.23
Hypertension	0.8865784 (0.5183912, 1.5162700)	0.66
Hemoglobin	0.9744476 (0.9620449, 0.9870103)	*<0.001*
Alb	0.8674282 (0.8141866, 0.9241514)	*<0.001*
eGFR	0.9779109 (0.9655423, 0.9904378)	*<0.01*
FPG	1.1219660 (1.0124180, 1.2433680)	*<0.05*
HbA1c	1.2742450 (1.0220770, 1.5886280)	*<0.05*
cTNT	1.0953920 (0.8179942, 1.4668600)	0.54
TC	1.0374960 (0.8384569, 1.2837850)	0.74
TG	0.9669338 (0.7421804, 1.2597490)	0.80
LDL-C	1.0332420 (0.7431301, 1.4366110)	0.85
HDL-C	0.5222179 (0.2337020, 1.1669200)	0.11
hs-CRP	1.0210930 (1.0052670, 1.0371690)	*<0.01*
ln(sFRP2)	0.5360921 (0.3668849, 0.7833375)	*<0.01*

*Statistically significant values are indicated in italic. BMI, body mass index; Alb, albumin; eGFR, estimated glomerular filtration rate; FPG, fasting plasma glucose; HbA1c, glycated hemoglobin; cTNT, cardiac troponin T; TC, total cholesterol; TG, triglyceride; LDL-C, low-density lipoprotein cholesterol; HDL-C, high-density lipoprotein cholesterol; hs-CRP, high-sensitivity C-reactive protein*.

**Table 6:** Risk factors for HF in 244 cardiac patients: multivariate logistic regression.

	**OR (95% CI)**	***P*-value**
Hemoglobin	0.9856071 (0.9714623, 0.9999579)	*<0.01*
Alb	0.9123722 (0.8501815, 0.9791121)	*<0.05*
eGFR	0.9835357 (0.9700838, 0.9971742)	*<0.01*
ln(sFRP2)	0.6633438 (0.4362906, 0.9985590)	*<0.05*

*Statistically significant values are indicated in italic. Alb, albumin; eGFR, estimated glomerular filtration rate*.

## Discussion

In our study, sFRP2 decreased significantly in the HF+T2DM group compared with the control group and the pre-clinical HF+T2DM group. Serum sFRP2 was negatively correlated with cTNT, NT-proBNP, LAD, LVPW, and positively correlated with LVEF. In addition, higher sFRP2 was significantly associated to lower odds of HF in patients with CVDs. These results suggest that the decreased circulating sFRP2 may be a risk factor for HF. Several experimental studies supported our observations and revealed the potential mechanisms. sFRP2 attenuated cardiac fibrosis and HF induced by pressure overload (19) and autoimmune myocarditis (16) *via* the Wnt/β-catenin and TGF-β pathways, respectively. sFRP2 prevented cardiomyocyte apoptosis (21) and pathological hypertrophy (19). *SFRP2* transgene mice exhibited higher LVEF and reduced infarct size due to the increased angiogenesis mediated by ATF6 signaling (20). In cardiac regeneration therapy, sFRP2 also improved bone marrow stromal cells (BMSCs) transplantation (22, 23) and enhanced the differentiation of cardiac progenitor cells (CPCs) (47). However, Yang *et al*. reported that in HF patients, sFRP2 was positively associated with extracellular volume (ECV) fraction, a parameter in cardiovascular magnetic resonance (CMR) imaging used to assess myocardial fibrosis. During follow-up periods, patients with primary cardiac events had higher levels of sFRP2 (25). Since they only enrolled patients with severe decompensated HF and NYHA functional class III–IV, the disparity may be attributed to different stages of HF and the elevated sFRP2 in their study may be a compensatory factor to counteract myocardial fibrosis. Indeed, an initial compensatory increase of cardiac sFRP2 level followed by a substantial decrease of sFRP2 during the progression of HF has already been reported in an animal model (19).

Our study also revealed that sFRP2 was lower in the HF+T2DM group than in the HF group and was negatively correlated with FPG and HbA1c. A previous study showed higher sFRP2 levels in patients with AGT (28). Since the diabetic patients in our study was subdivided from patients with CVDs, the effect of CVDs on sFRP2 may be the main reason for the difference. This discrepancy may also be attributed to their enrollment of pre-diabetes patients. Prediabetes is also associated with increased risk of CVDs (48) and HF (49). Further studies are needed to explore whether sFRP2 is associated with prediabetes and whether this association, if any, is involved in CVDs and HF.

It is intriguing that serum sFRP2 levels reduced in the co-occurrence of HF and T2DM, rather than in either individual disease. We performed a univariate logistic regression analysis specifically in patients with CVDs and T2DM (the pre-clinical HF+T2DM group and the HF+T2DM group, data not shown), and found a more obvious inverse association between ln(sFRP2) and HF (OR: 0.42, 95% CI: 0.24–0.74, *P* < 0.01) compared with that in all the patients with CVDs (OR: 0.54, 95% CI: 0.37–0.78, *P* < 0.05). Patients with the comorbidity of HF and T2DM have distinct biomarkers compared to those without T2DM (50) and sFRP2 is potentially one of them. A falling heart undergoes metabolic perturbations characterized by impaired lipid metabolism and a substrate preference switch toward glycolysis and ketone body oxidation (51). The hyperglycemia, insulin resistance, and hyperinsulinemia in T2DM trigger a cascade of deleterious effects that contribute to the development of HF (52). sFRP2 was reported to be associated with lipogenesis and insulin resistance (53), which indicated that sFRP2 might modulate heart function *via* regulating metabolism.

In our study, the level of hs-CRP in the HF+T2DM group was higher than that in other groups and sFRP2 was negatively correlated with hs-CRP. This is unsurprising since HF and T2DM are accompanied by chronic low-grade systemic inflammation (54). Previous studies have reported the association between sFRP2 and inflammation. sFRP2 was downregulated in inflammation-induced muscle atrophy and prevented inflammatory muscle atrophy (55). sFRP2 enhanced the osteo/odontogenic differentiation and paracrine potentials under inflammation conditions *via* inhibiting canonical Wnt/β-catenin and nuclear factor kappa B (NF-kB) signaling pathways (56). However, Zhou *et al*. demonstrated that serum sFRP2 was significantly upregulated in patients with COPD and knockdown of sFRP2 in peripheral blood mononuclear cells (PBMCs) attenuated airway inflammation (57). Thus, further studies are mandated to explore the detailed roles of sFRP2 in cardiovascular inflammation.

The main strengths of our study are the inclusion of people with different HF stages, which allow us to explore the association of sFRP2 and severity of HF. Furthermore, we collected and adjusted multiple cardiovascular risk factors. However, several limitations should be noted. First, the cross-sectional design precluded us from drawing casual conclusions and further studies are required to define the role of sFRP2 in cardiac function and glucose homeostasis. Second, there were significant differences in etiologies between each group, although there were no significant differences of sFRP2 between ischemic and non-ischemic heart diseases in each group (data not shown) and Spearman rank correlation analysis confirmed no significant association between sFRP2 and etiologies. Third, the measurement of serum sFRP2 by ELISA could not reflect its sources and targets. *SFRP2* gene is ubiquitously expressed in adipose tissue, small intestine, colon, heart, skeletal muscle and other organs, with the highest expression in adipose tissue (58, 59). As an adipokine, circulating sFRP2 is not linearly correlated with *SFRP2* gene expression in WAT (27), indicating that other sources of sFRP2 production should be investigated. Since the cardiac sFRP2 expression increased in the early stage of HF and subsequently decreased with the development of HF (19), less sFRP2 production from heart may explain the declined circulating sFRP2. Previous studies focused on the autocrine and paracrine function, while the changes in serum sFRP2 may indicate its endocrine function. SFRP2 exerts effects on cardiomyocytes, cardiac fibroblast cells, endothelial cells (24) and adipocytes (28) and its function exhibits a high degree of cell specificity. More researches are necessary to explore why serum sFRP2 reduced in the comorbidity of HD and T2DM and the relevant mechanism.

## Conclusions

The level of sFRP2 was negatively correlated with age, FPG, HbA1c, cTNT, NT-proBNP, hs-CRP, LAD and LVPW, and positively correlated with hemoglobin, eGFR, albumin, TC, LDL-C and LVEF. Higher serum sFRP2 was significantly linked to lower odds of HF in patients with CVDs. sFRP2 is a promising risk factor for the comorbidity of HF and T2DM, which might pave novel ways for the diagnosis and treatment of clinical HF from the perspective of metabolism.

## Data Availability Statement

The raw data supporting the conclusions of this article will be made available by the authors, without undue reservation.

## Ethics Statement

The studies involving human participants were reviewed and approved by Ethics Committee of Zhongshan Hospital affiliated to Fudan University. The patients/participants provided their written informed consent to participate in this study.

## Author Contributions

PG and MC designed the study. PG, MC, HW, and XW performed the experiments. WL and XJ contributed to the interpretation of the results. MC analyzed the data and wrote the first draft of the manuscript. WG, PG, and YZ supervised the project and revised the paper. All authors contributed to the article and approved the submitted version.

## Funding

This work was supported by Shanghai Rising-Star Program (21QA1401700), National Natural Science Foundation of China (82000271 and 81730009), and Scientific Research Project of Shanghai Health Commission (20204Y0225).

## Conflict of Interest

The authors declare that the research was conducted in the absence of any commercial or financial relationships that could be construed as a potential conflict of interest.

## Publisher's Note

All claims expressed in this article are solely those of the authors and do not necessarily represent those of their affiliated organizations, or those of the publisher, the editors and the reviewers. Any product that may be evaluated in this article, or claim that may be made by its manufacturer, is not guaranteed or endorsed by the publisher.

## References

[1] Murphy SP Ibrahim NE Januzzi JL Jr. Heart failure with reduced ejection fraction: a review. Jama. (2020) 324:488–504. 10.1001/jama.2020.10262 32749493

[2] Hunt SA Baker DW Chin MH Cinquegrani MP Feldman AM Francis GS. ACC/AHA guidelines for the evaluation and management of chronic heart failure in the adult: executive summary a report of the American College of Cardiology/American Heart Association Task Force on practice guidelines (committee to revise the 1995 guidelines for the evaluation and management of heart failure): developed in collaboration with the international society for heart and lung transplantation; endorsed by the Heart Failure Society of America. Circulation. (2001) 104:2996–3007. 10.1161/hc4901.102568 11739319

[3] Yancy CW Jessup M Bozkurt B Butler J Casey DE Jr Drazner MH. 2013 ACCF/AHA guideline for the management of heart failure: a report of the American College of Cardiology Foundation/American Heart Association Task Force on Practice Guidelines. J Am Coll Cardiol. (2013) 62:e147–239. 10.1016/j.jacc.2013.05.019 23747642

[4] Chinese Society of Cardiology. Guidelines for the diagnosis and management of chronic heart failure. Chin J Cardiovasc Med. (2007) 35:1076–95. 10.3760/j.issn:0253-3758.2007.12.002 18341806

[5] Ammar KA Jacobsen SJ Mahoney DW Kors JA Redfield MM Burnett JC Jr. Prevalence and prognostic significance of heart failure stages: application of the American College of Cardiology/American Heart Association heart failure staging criteria in the community. Circulation. (2007) 115:1563–70. 10.1161/CIRCULATIONAHA.106.666818 17353436

[6] Dunlay SM Givertz MM Aguilar D Allen LA Chan M Desai AS. Type 2 diabetes mellitus and heart failure: a scientific statement from the american heart association and the heart failure society of america: this statement does not represent an update of the 2017 ACC/AHA/HFSA heart failure guideline update. Circulation. (2019) 140:e294–324. 10.1161/CIR.0000000000000691 31167558

[7] Li Y Teng D Shi X Qin G Qin Y Quan H. Prevalence of diabetes recorded in mainland China using 2018 diagnostic criteria from the American Diabetes Association: national cross sectional study. BMJ. (2020) 369:m997. 10.1136/bmj.m997 32345662 PMC7186854

[8] Kannel WB McGee DL. Diabetes and cardiovascular disease. The Framingham study. JAMA. (1979) 241:2035–8. 10.1001/jama.241.19.2035 430798

[9] Nichols GA Gullion CM Koro CE Ephross SA Brown JB. The incidence of congestive heart failure in type 2 diabetes: an update. Diabetes Care. (2004) 27:1879–84. 10.2337/diacare.27.8.1879 15277411

[10] van Melle JP Bot M de Jonge P de Boer RA van Veldhuisen DJ Whooley MA. Diabetes, glycemic control, and new-onset heart failure in patients with stable coronary artery disease: data from the heart and soul study. Diabetes Care. (2010) 33:2084–9. 10.2337/dc10-0286 20805280 PMC2928369

[11] Iribarren C Karter AJ Go AS Ferrara A Liu JY Sidney S. Glycemic control and heart failure among adult patients with diabetes. Circulation. (2001) 103:2668–73. 10.1161/01.CIR.103.22.2668 11390335

[12] Pazin-Filho A Kottgen A Bertoni AG Russell SD Selvin E Rosamond WD. HbA 1c as a risk factor for heart failure in persons with diabetes: the Atherosclerosis Risk in Communities (ARIC) study. Diabetologia. (2008) 51:2197–204. 10.1007/s00125-008-1164-z 18828004 PMC2848756

[13] Cavender MA Steg PG Smith SC Jr Eagle K Ohman EM Goto S. Impact of diabetes mellitus on hospitalization for heart failure, cardiovascular events, and death: outcomes at 4 years from the reduction of atherothrombosis for continued health (REACH) registry. Circulation. (2015) 132:923–31. 10.1161/CIRCULATIONAHA.114.014796 26152709

[14] Huang A Huang Y. Role of Sfrps in cardiovascular disease. Ther Adv Chronic Dis. (2020) 11:2040622320901990. 10.1177/2040622320901990 32064070 PMC6987486

[15] Aubert J Dunstan H Chambers I Smith A. Functional gene screening in embryonic stem cells implicates Wnt antagonism in neural differentiation. Nat Biotechnol. (2002) 20:1240–5. 10.1038/nbt763 12447396

[16] Blyszczuk P Müller-Edenborn B Valenta T Osto E Stellato M Behnke S. Transforming growth factor-β-dependent Wnt secretion controls myofibroblast formation and myocardial fibrosis progression in experimental autoimmune myocarditis. Eur Heart J. (2017) 38:1413–25. 10.1093/eurheartj/ehw116 27099262

[17] Lin H Angeli M Chung KJ Ejimadu C Rosa AR Lee T. sFRP2 activates Wnt/beta-catenin signaling in cardiac fibroblasts: differential roles in cell growth, energy metabolism, and extracellular matrix remodeling. Am J Physiol Cell Physiol. (2016) 311:C710–9. 10.1152/ajpcell.00137.2016 27605451 PMC5130588

[18] Hao K Lei W Wu H Wu J Yang Z Yan S. LncRNA-Safe contributes to cardiac fibrosis through Safe-Sfrp2-HuR complex in mouse myocardial infarction. Theranostics. (2019) 9:7282–97. 10.7150/thno.33920 31695768 PMC6831303

[19] Wei WY Zhao Q Zhang WZ Wang MJ Li Y Wang SZ. Secreted frizzled-related protein 2 prevents pressure-overload-induced cardiac hypertrophy by targeting the Wnt/β-catenin pathway. Mol Cell Biochem. (2020) 472:241–51. 10.1007/s11010-020-03802-x 32632611 PMC7338134

[20] Vatner DE Oydanich M Zhang J Babici D Vatner SF. Secreted frizzled-related protein 2, a novel mechanism to induce myocardial ischemic protection through angiogenesis. Basic Res Cardiol. (2020) 115:48. 10.1007/s00395-020-0808-0 32592071 PMC8530433

[21] Zhang Z Deb A Zhang Z Pachori A He W Guo J. Secreted frizzled related protein 2 protects cells from apoptosis by blocking the effect of canonical Wnt3a. J Mol Cell Cardiol. (2009) 46:370–7. 10.1016/j.yjmcc.2008.11.016 19109969 PMC2710029

[22] Lin M Liu X Zheng H Huang X Wu Y Huang A. IGF-1 enhances BMSC viability, migration, and anti-apoptosis in myocardial infarction via secreted frizzled-related protein 2 pathway. Stem Cell Res Ther. (2020) 11:22. 10.1186/s13287-019-1544-y 31918758 PMC6953226

[23] Mirotsou M Zhang Z Deb A Zhang L Gnecchi M Noiseux N. Secreted frizzled related protein 2 (Sfrp2) is the key Akt-mesenchymal stem cell-released paracrine factor mediating myocardial survival and repair. Proc Natl Acad Sci U S A. (2007) 104:1643–8. 10.1073/pnas.0610024104 17251350 PMC1785280

[24] Wu Y Liu X Zheng H Zhu H Mai W Huang X. Multiple roles of sFRP2 in cardiac development and cardiovascular disease. Int J Biol Sci. (2020) 16:730–8. 10.7150/ijbs.40923 32071544 PMC7019133

[25] Yang S Chen H Tan K Cai F Du Y Lv W. Secreted frizzled-related protein 2 and extracellular volume fraction in patients with heart failure. Oxid Med Cell Longev. (2020) 2020:2563508. 10.1155/2020/2563508 32454934 PMC7229555

[26] Ehrlund A Mejhert N Lorente-Cebrián S Aström G Dahlman I Laurencikiene J. Characterization of the Wnt inhibitors secreted frizzled-related proteins (SFRPs) in human adipose tissue. J Clin Endocrinol Metab. (2013) 98:E503–8. 10.1210/jc.2012-3416 23393180

[27] Schübel R Sookthai D Greimel J Johnson TS Grafetstätter ME Kirsten R. Key genes of lipid metabolism and WNT-signaling are downregulated in subcutaneous adipose tissue with moderate weight loss. Nutrients. (2019) 11:639. 10.3390/nu11030639 30884788 PMC6471921

[28] Crowley RK O'Reilly MW Bujalska IJ Hassan-Smith ZK Hazlehurst JM Foucault DR. SFRP2 is associated with increased adiposity and vegf expression. PLoS ONE. (2016) 11:e0163777. 10.1371/journal.pone.0163777 27685706 PMC5042473

[29] Baldane S Ipekci SH Ekin A Abusoglu S Unlu A Kebapcilar L. Evaluation of fractalkine (FKN) and secreted frizzled-related protein 4 (SFRP-4) serum levels in patients with prediabetes and type 2 diabetes. Bratisl Lek Listy. (2018) 119:112–5. 10.4149/BLL_2018_021 29455547

[30] Bukhari SA Yasmin A Zahoor MA Mustafa G Sarfraz I Rasul A. Secreted frizzled-related protein 4 and its implication in obesity and type-2 diabetes. IUBMB Life. (2019) 71:1701–10. 10.1002/iub.2123 31301214

[31] Baldane S Ipekci SH Kebapcilar AG Abusoglu A Beyhekim H Ilhan TT. Prorenin and secreted frizzled-related protein 4 levels in women with gestational diabetes mellitus. Bratisl Lek Listy. (2018) 119:450–3. 10.4149/BLL_2018_083 30160136

[32] Senyigit A Uzun H Gultepe I Konukoglu D. The relationship between carotid intima-media thickness and serum secreted frizzled-related protein-4 and dipeptidyl peptidase-4 in diabetic patients with cardiovascular diseases. Bratisl Lek Listy. (2019) 120:188–94. 10.4149/BLL_2019_032 31023036

[33] Carstensen-Kirberg M Kannenberg JM Huth C Meisinger C Koenig W Heier M. Inverse associations between serum levels of secreted frizzled-related protein-5 (SFRP5) and multiple cardiometabolic risk factors: KORA F4 study. Cardiovasc Diabetol. (2017) 16:109. 10.1186/s12933-017-0591-x 28851362 PMC5574239

[34] Oztas E Ozler S Ersoy E Ersoy AO Tokmak A Ergin M. Prediction of gestational diabetes mellitus by first trimester serum secreted frizzle-related protein-5 levels. J Matern Fetal Neonatal Med. (2016) 29:1515–9. 10.3109/14767058.2015.1052399 26100762

[35] Hu Z Deng H Qu H. Plasma SFRP5 levels are decreased in Chinese subjects with obesity and type 2 diabetes and negatively correlated with parameters of insulin resistance. Diabetes Res Clin Pract. (2013) 99:391–5. 10.1016/j.diabres.2012.11.026 23290274

[36] Miyoshi T Doi M Usui S Iwamoto M Kajiya M Takeda K. Low serum level of secreted frizzled-related protein 5, an anti-inflammatory adipokine, is associated with coronary artery disease. Atherosclerosis. (2014) 233:454–9. 10.1016/j.atherosclerosis.2014.01.019 24530778

[37] Wu J Zheng H Liu X Chen P Zhang Y Luo J. Prognostic value of secreted frizzled-related protein 5 in heart failure patients with and without type 2 diabetes mellitus. Circ Heart Fail. (2020) 13:e007054. 10.1161/CIRCHEARTFAILURE.120.007054 32842761

[38] Chinese Society of Cardiology. Guidelines for the diagnosis and management of heart failure 2018. Chin J Cardiovasc Med. (2018) 46:760–89. 10.3760/cma.j.issn.0253-3758.2018.10.004 30369168

[39] Chinese Diabetes Society. Guideline for the prevention and treatment of type 2 diabetes mellitus in China (2020 edition). Chin J Diabetes Mellitus. (2021) 13:315–409. 10.3760/cma.j.cn115791-20210221-00095

[40] Chinese Society of Cardiology Chinese Geriatrics Society. 2018 Chinese guidelines for the management of hypertension. Chin J Cardiovasc Med. (2019) 24:24–56. 10.3969/j.issn.1007-5410.2019.01.002

[41] Chinese Society of Cardiology. Guidelines for the diagnosis and management of stable coronary artery disease. Chin J Cardiovasc Med. (2018) 46:680–94. 10.3760/cma.j.issn.0253-3758.2018.09.004 30293374

[42] Chinese Society of Cardiology. 2016 Guidelines for the diagnosis and management of non-ST-segment elevation acute coronary syndrome. Chin J Cardiovasc Med. (2017) 45:359–76. 10.3760/cma.j.issn.0253-3758.2017.05.003 28511320

[43] Otto CM Nishimura RA Bonow RO Carabello BA Erwin JP III Gentile F. 2020 ACC/AHA guideline for the management of patients with valvular heart disease: executive summary: a report of the american college of cardiology/american heart association joint committee on clinical practice guidelines. Circulation. (2021) 143:e35–71. 10.1161/CIR.0000000000000932 33332149

[44] Chinese Society of Cardiology. Guidelines for the diagnosis and management of adult hypertrophic cardiomyopathy. Chin J Cardiovasc Med. (2017) 45:1015–32. 10.3760/cma.j.issn.0253-3758.2017.12.005 29325361

[45] Baumgartner H De Backer J Babu-Narayan SV Budts W Chessa M Diller GP. 2020 ESC Guidelines for the management of adult congenital heart disease. Eur Heart J. (2021) 42:563–645. 10.1093/eurheartj/ehaa554 32860028

[46] Yancy CW Jessup M Bozkurt B Butler J Casey DE Jr Colvin MM. 2017 ACC/AHA/HFSA Focused Update of the 2013 ACCF/AHA Guideline for the Management of Heart Failure: a report of the American College of Cardiology/American Heart Association Task Force on Clinical Practice Guidelines and the Heart Failure Society of America. Circulation. (2017) 136:e137–61. 10.1161/CIR.0000000000000509 28455343

[47] Schmeckpeper J Verma A Yin L Beigi F Zhang L Payne A. Inhibition of Wnt6 by Sfrp2 regulates adult cardiac progenitor cell differentiation by differential modulation of Wnt pathways. J Mol Cell Cardiol. (2015) 85:215–25. 10.1016/j.yjmcc.2015.06.003 26071893 PMC4838816

[48] Cai X Zhang Y Li M Wu JH Mai L Li J. Association between prediabetes and risk of all cause mortality and cardiovascular disease: updated meta-analysis. BMJ. (2020) 370:m2297. 10.1136/bmj.m2297 32669282 PMC7362233

[49] Cai X Liu X Sun L He Y Zheng S Zhang Y. Prediabetes and the risk of heart failure: a meta-analysis. Diabetes Obes Metab. (2021) 23:1746–53. 10.1111/dom.14388 33769672

[50] Sharma A Demissei BG Tromp J Hillege HL Cleland JG O'Connor CM. A network analysis to compare biomarker profiles in patients with and without diabetes mellitus in acute heart failure. Eur J Heart Fail. (2017) 19:1310–20. 10.1002/ejhf.912 28639369

[51] Bedi KC Jr Snyder NW Brandimarto J Aziz M Mesaros C Worth AJ. Evidence for intramyocardial disruption of lipid metabolism and increased myocardial ketone utilization in advanced human heart failure. Circulation. (2016) 133:706–16. 10.1161/CIRCULATIONAHA.115.017545 26819374 PMC4779339

[52] Marwick TH Ritchie R Shaw JE Kaye D. Implications of underlying mechanisms for the recognition and management of diabetic cardiomyopathy. J Am Coll Cardiol. (2018) 71:339–51. 10.1016/j.jacc.2017.11.019 29348027

[53] Kuppusamy P Ilavenil S Hwang IH Kim D Choi KC. Ferulic acid stimulates adipocyte-specific secretory proteins to regulate adipose homeostasis in 3T3-L1 adipocytes. Molecules. (2021) 26:1984. 10.3390/molecules26071984 33915783 PMC8037266

[54] Furman D Campisi J Verdin E Carrera-Bastos P Targ S Franceschi C. Chronic inflammation in the etiology of disease across the life span. Nat Med. (2019) 25:1822–32. 10.1038/s41591-019-0675-0 31806905 PMC7147972

[55] Zhu X Kny M Schmidt F Hahn A Wollersheim T Kleber C. Secreted frizzled-related protein 2 and inflammation-induced skeletal muscle atrophy. Crit Care Med. (2017) 45:e169–83. 10.1097/CCM.0000000000002056 27661566

[56] Yang H Li G Han N Zhang X Cao Y Cao Y. Secreted frizzled-related protein 2 promotes the osteo/odontogenic differentiation and paracrine potentials of stem cells from apical papilla under inflammation and hypoxia conditions. Cell Prolif. (2020) 53:e12694. 10.1111/cpr.12694 31568642 PMC6985663

[57] Zhou M Jiao L Liu Y. sFRP2 promotes airway inflammation and Th17/Treg imbalance in COPD via Wnt/β-catenin pathway. Respir Physiol Neurobiol. (2019) 270:103282. 10.1016/j.resp.2019.103282 31430541

[58] Hu E Zhu Y Fredrickson T Barnes M Kelsell D Beeley L. Tissue restricted expression of two human Frzbs in preadipocytes and pancreas. Biochem Biophys Res Commun. (1998) 247:287–93. 10.1006/bbrc.1998.8784 9642118

[59] Melkonyan HS Chang WC Shapiro JP Mahadevappa M Fitzpatrick PA Kiefer MC. SARPs: a family of secreted apoptosis-related proteins. Proc Natl Acad Sci U S A. (1997) 94:13636–41. 10.1073/pnas.94.25.13636 9391078 PMC28358

